# Probing Xist RNA Structure in Cells Using Targeted Structure-Seq

**DOI:** 10.1371/journal.pgen.1005668

**Published:** 2015-12-08

**Authors:** Rui Fang, Walter N. Moss, Michael Rutenberg-Schoenberg, Matthew D. Simon

**Affiliations:** 1 Department of Molecular Biophysics & Biochemistry, Yale University, New Haven, Connecticut, United States of America; 2 Chemical Biology Institute, Yale University, West Haven, Connecticut, United States of America; Los Alamos National Laboratory, UNITED STATES

## Abstract

The long non-coding RNA (lncRNA) Xist is a master regulator of X-chromosome inactivation in mammalian cells. Models for how Xist and other lncRNAs function depend on thermodynamically stable secondary and higher-order structures that RNAs can form in the context of a cell. Probing accessible RNA bases can provide data to build models of RNA conformation that provide insight into RNA function, molecular evolution, and modularity. To study the structure of Xist in cells, we built upon recent advances in RNA secondary structure mapping and modeling to develop Targeted Structure-Seq, which combines chemical probing of RNA structure in cells with target-specific massively parallel sequencing. By enriching for signals from the RNA of interest, Targeted Structure-Seq achieves high coverage of the target RNA with relatively few sequencing reads, thus providing a targeted and scalable approach to analyze RNA conformation in cells. We use this approach to probe the full-length Xist lncRNA to develop new models for functional elements within Xist, including the repeat A element in the 5’-end of Xist. This analysis also identified new structural elements in Xist that are evolutionarily conserved, including a new element proximal to the C repeats that is important for Xist function.

## Author Summary

To do their jobs, many RNAs need to fold into structures (through base-paring). We were interested in the conformation of a specific mammalian RNA, Xist, when it is inside a cell. Xist is a very large non-coding RNA (lncRNA), that is >17,000 nt long. Xist is particularly important because it is one of the first lncRNAs to be discovered, and turns genes off across an entire chromosome. To figure out how Xist RNA is folded in mouse cells, we developed a new approach, Targeted Structure-Seq, to examine the conformation of large RNAs like Xist. Using computer modeling, we identified parts of Xist that are base paired into RNA duplexes. We also determined which parts of the Xist RNA are likely to be structured. This work provides a new tool for studying the secondary structure of any large RNA, and helps us understand what the important pieces of Xist look like while Xist does its work in the cell.

## Introduction

One of the earliest discoveries of a mammalian long non-coding RNA (lncRNA) with a well-defined role in gene regulation is the ∼18 kb Xist lncRNA (NR_001463.3) [1, 2]. Xist drives chromosome-wide repression of one of the two female X-chromosomes during mammalian development in order to balance gene dosage between males (XY) and females (XX) [3–6]. During early development in female embryos, Xist transcription is up-regulated on the future inactive X-chromosome. Xist spreads by a two-step mechanism [7] to eventually coat the majority of the X-chromosome [3–6, 8]. Xist directs dramatic and mitotically stable changes on the inactive X chromosome, including DNA methylation, histone methylation, and changes in histone variant incorporation. Despite the relatively long history of studying Xist compared to other lncRNAs, surprisingly little is known about the *in vivo* conformations of Xist or its molecular mechanism [9].

**Fig 1:**
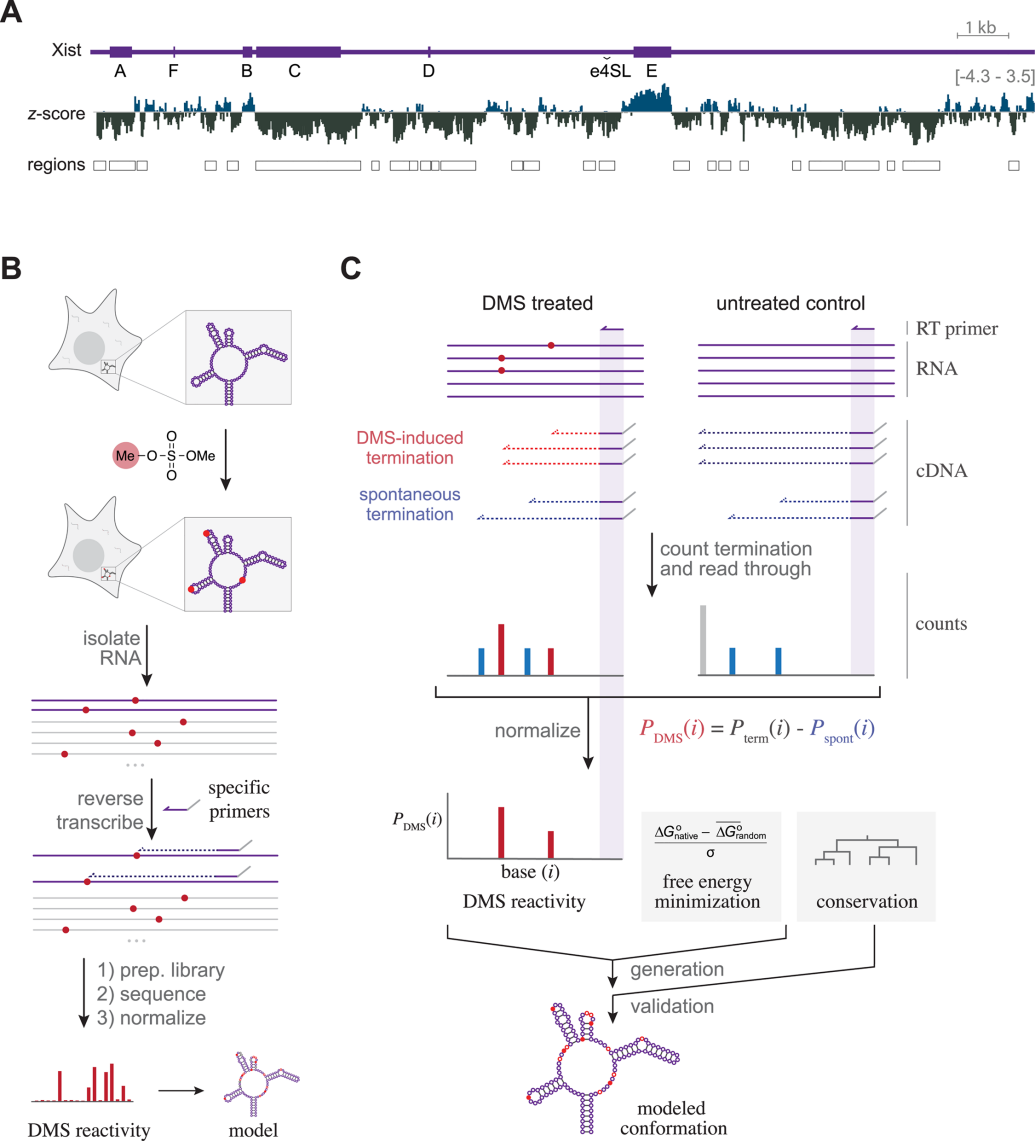
Examining the conformations of elements within Xist RNA using a mixture of computational approaches and Targeted Structure-Seq. **A.** Xist RNA (purple) contains several repetitive elements (indicated as thick regions, labeled A-F), and a conserved stem-loop structure in exon 4 (labeled as e4SL). The predicted *z*-score for 150 nt windows calculated every 10 nt (negative and positive values in gray and blue, respectively). Boxes indicate overlapping windows with *z*-score <1*σ* Xist average, classified as low *z*-score regions. **B.** An overview of Targeted Structure-Seq. A target RNA (purple) can be methylated (red circles) with dimethylsulfate (DMS, shown with red methyl group) in cells. After RNA isolation, the sites of methylation are determined using reverse transcription with primers specific for the RNA of interest (purple) but not other RNAs (gray). Sequencing and analysis of these data can be used to determine the DMS reactivity at each base, and can aid in modeling of the cellular conformation of the RNA. **C.** To determine which termination events were caused by DMS (red) as opposed to spontaneous termination (blue), the termination and read-through events are counted. Using an untreated control to estimate the rate of spontaneous termination, the data is normalized to determine the DMS reactivity at each base (see Materials and Methods for details). These data can be used for free energy minimization to model the RNA conformation, which can be further validated by examining the evolutionary conservation of base pairing.

Xist has at least two partially separable functions: silencing of transcription, and spreading on the X chromosome. These functions map to different features within the RNA [10]. The most notable feature of the Xist RNA is a set of diverse regions that include distinct repeat sequences, denoted A-F (Fig 1A), several of which show conservation across species [1, 2]. Each of these repetitive elements have a different arrangement; for example, the C-repeat region (3098-4712 nt) is composed of 14 copies of a ∼120 nt monomer, while the A-repeat region, located near the 5’ terminus (308-733 nt), is composed of 7.5 repeats of a ∼25 nt monomer consensus sequence connected by poorly conserved, 17-49 nt U-rich linker sequences. The A-repeat region is the most well-studied structural element in Xist, and is necessary and sufficient to cause transcriptional silencing in mouse and human [10, 11]. This motif has been reported to interact with various protein factors including the polycomb repressive complex 2 (PRC2) [12, 13] and the ASF/SF2 splicing factors [14]. While deletion analyses have uncovered evidence for other functional elements, the regions of Xist required for spreading and recruitment of other protein factors are still poorly defined, at least in part because of redundancy within the functional motifs [10]. Nonetheless, it is noteworthy that short antisense peptide nucleic acid (PNA) and locked nucleic acid (LNA) oligonucleotides targeting the repeat C region of Xist can cause release of Xist from the chromosome without RNA degradation. [15, 16] These findings support the notion that the C repeat region of Xist is involved in maintaining the association of the RNA with the chromatin. It remains largely unclear how many functional elements will be found within Xist, where they are located, and how they are structured.

Understanding the cellular functions of RNA features within Xist would be greatly assisted by knowing their *in vivo* conformations. RNA elements, similar to peptide motifs, can fold into thermodynamically stable structures to fulfill their regulatory roles. Our ability to predict RNA secondary structure using minimal free energy is limited by the size of the RNA element of interest as well as the availability and diversity of sequences for comparative sequence analysis. Experimental data that discriminate paired and unpaired bases in an RNA molecule can greatly increase prediction accuracy [17]. Therefore, building accurate models of the conformations of elements within Xist would be greatly facilitated by systematic probing of its structure in the context of a cell.

The conformations of two regions of Xist have been systematically probed: the repeat A region and a conserved stem-loop structure found in exon 4 of Xist. The repeat A region of murine and human Xist has been examined by comparing predicted thermodynamically stable structures to data from *in vitro* analyses [4, 10, 18, 19]. A stem loop in exon 4 that has been investigated [20] was confirmed to be structured *in vitro* and in permeabilized nuclei [21]. The conformation of the remainder of Xist has not been systematically probed, nor is it clear how the *in vitro* configuration of Xist relates to its conformation in cells. Addressing these questions would require a scalable means of probing RNA conformation in cells.

Prior to this study, no approach existed that allowed targeted analysis of the conformation of large RNAs such as Xist in mammalian cells; however, there have been several recent advances in RNA structural probing that pave the way for such a technique. Dimethyl sulfate (DMS) is one of the few chemicals that have been successfully used to probe RNA structure *in vivo*. DMS is cell-permeable and rapidly alkylates unpaired or loosely structured A and C bases in folded RNAs. The modified bases can be identified by the termination of reverse-transcription (RT) at the site of a modified base. Traditionally, the readout of sites of DMS modification was performed using electrophoresis (either slab gel or capillary formats), limiting the scale of analysis [22]. The scalability of these chemical probing experiments has been dramatically increased by the use of massively parallel sequencing technology [23, 24]. Indeed, experiments such as DMS-Seq [25] and Structure-Seq [26] are capable of acquiring *in vivo* DMS data transcriptome-wide. However, these technologies target the whole genome, compromising the depth of probing (and therefore accuracy of determining modified bases [27]) of any specific RNA. While targeted *in vivo* approaches using DMS have been pioneered (*e.g.*, see Kwok, *et al.* [28]), these generally rely on electrophoresis, thereby limiting the scale of analysis. ChemMod-Seq is an encouraging approach as it allowed chemical probing of ribosomal and pre-ribosomal RNAs in yeast, and coupled this probing to a sequencing assay [29]. To adapt such an approach to probe Xist conformations in cells, we sought an approach that combines the depth and accuracy of on-target approaches with the scalability of experiments that use a sequencing platform to allow systematic analysis of an ∼18 kb lncRNA.

In order to generate experimental constraints to provide accurate models of lncRNA conformation in mammalian cells, we developed and validated Targeted Structure-Seq. Similar to ChemMod-Seq [29], this in-cell method combines the advantages of on-target DMS probing [28] with the scalability of a sequencing platform [23–26]. We validated the enrichment, reproducibility and robustness of the approach, and confirmed expected base reactivities using the 18S rRNA. This approach allowed us to probe full-length Xist RNA in mouse cells. This analysis provides the first in-cell model of the Xist repeat A conformation and identifies new structured elements in Xist, including a new evolutionary conserved structural element near the C repeats that is important for Xist function.

## Results

### *In silico* scan of Xist for regions of RNA secondary structure

While chemical probing experiments increase the accuracy of modeling RNA conformation, we first used a strictly computational approach to ask which elements within Xist are likely to be structured [30]. Predicting the conformation of a large RNA such as Xist presents significant challenges. Most known structured RNAs are relatively small, and prediction accuracy is highest for sequences shorter than ∼500 nucleotides [31, 32]. We were interested in identifying regions of Xist that are likely to form stable RNA structures, without limiting our analysis to fragments of fixed sizes.

Structured RNA is expected to have lower minimal free energy of folding compared to randomized sequence of the same composition [33]. We scanned the full-length Xist RNA using a sliding window (150 nt window with 10 nt steps) and calculated the predicted folding free energy of each window relative to randomized nucleotides of the same composition using RNAfold [34, 35]. Thermodynamic *z*-scores were calculated to measure how much more stable (number of standard deviations) the native sequence was relative to random sequences. More negative *z*-scores suggest that a sequence evolved to maintain a particular secondary structure [33]. The *z*-score depends on the sequence and, to a lesser degree, its composition (*e.g.*, GC%). As *z*-scores provide the basis for programs that have successfully predicted functional non-coding RNAs [36, 37], we used them to uncover regions in Xist that may be structured.

Based on these predictions, Xist displays a moderate overall propensity for RNA structure (average *z*-score per 150 nt window = -0.83). However, certain windows within Xist have exceptionally low *z*-scores, indicating that these regions have a high propensity for forming RNA structure (Fig 1A). For example, the window with the lowest *z*-score (6661–6810 nt) is over four standard deviations more stable than random sequences of the same composition (*z*-score = -4.41). To define likely structural domains, we combined overlapping windows with *z*-scores below a strict cutoff of -2.19 (1 *σ* more negative than Xist average) and predicted that 26 regions of murine Xist (spanning a total of 9360 nt) are structured (Table 1). These regions average 360 nt and range from 150 nucleotides (a single isolated window) to 1990 nucleotides, and include many elements that may be important for Xist function.

**Table 1:** Xist regions with exceptionally favorable *z*-scores^1^.

position		*z*-score		$\Delta G_{calc}^{\circ }$		conservation	
start	end	average	minimum	average	minimum	rodents	mammals
1	240	-2.81	-3.72	-26.48	-32.86	+	
301	790^*a*^	-1.85	-3.69	-23.65	-34.30	+	+
831	1020	-2.28	-2.73	-43.84	-46.97	+	
2141	2350	-2.25	-3.12	-32.31	-33.84	+	+/-
2551	2770	-1.68	-2.75	-28.57	-31.44	+	
3101	5090^*c*^	-2.15	-4.05	-21.36	-31.75	+/-	+/-
5301	5450	-2.88	—	-25.02	—	+	
5661	6020	-2.54	-3.46	-30.65	-34.41	+	
6031	6180	-2.53	—	-24.28	—	+	+
6231	6430^*d*^	-2.07	-2.34	-28.77	-29.88	+	
6441	6590	-2.47	—	-18.42	—	+	+/-
6631	7300	-2.14	-4.41	-25.37	-30.86	+	+
7981	8180	-2.32	-2.92	-20.25	-22.84	+	+/-
8201	8500	-2.38	-3.27	-31.13	-35.55	+	+
9351	9580	-2.35	-2.75	-30.09	-33.07	+	+
9651	9950	-1.65	-2.88	-39.31	-47.24	+	+
11071	11360	-1.60	-2.51	-16.23	-20.65	+	
11721	11880	-2.48	-2.75	-31.86	-32.47	+	
11921	12150	-1.78	-3.30	-24.68	-25.62	+	
12331	12490	-3.41	-3.56	-29.24	-29.87	+	+
13321	13490	-1.87	-2.45	-28.57	-30.03	+	+/-
13651	14270	-2.14	-4.01	-28.56	-33.47	+	+/-
14321	14970	-2.03	-3.66	-26.23	-33.83	+	+/-
15131	15280	-2.28	—	-26.10	—	+	
15431	16130	-2.35	-3.95	-26.88	-23.96	+	+/-
17461	17640	-2.38	-2.64	-27.32	-27.34	+	+
1	17918^*x*^	-0.83	-	-27.14	-	

^1^Minimal free energies of Xist RNA fragments and randomized sequences were calculated in 150 nt sliding windows in 10 nt steps. Regions were identified as binned windows with a *z*-score less than 1*σ* below Xist average. The *z*-score and predicted folding free energies ($\Delta G_{calc}^{\circ }$) of each region (average) and the most favorable window (minimal) are shown.

^*a*^repeat A region

^*c*^includes C repeats

^*d*^overlaps with D repeats

^*x*^average of full-length Xist

The 26 low *z*-score regions are distributed across Xist. Some regions correlate with repetitive sequence motifs (repeat A, C, and D; Fig 1A). For example, all A repeats (308-733 nt) are encompassed in a predicted structured region spanning 301–790 nt. The repeat C region is the largest repetitive element in Xist, and overlaps the largest low *z*-score region (nucleotides 3101–5090, see Fig 1), consistent with this region being tightly constrained by RNA structure. Conversely, there are distinct regions that correlate with other repetitive regions of Xist (*e.g.,* 10031-11080 nt in Fig 1, which includes the E repeats) that are predicted to have very *un*favorable (positive) *z*-scores, suggesting that these regions may be unusually unstructured [34, 38]. Both of the elements previously shown to have structural propensity in Xist (the A repeats and the hairpin in exon 4) are included within the regions defined by low *z*-scores (Table 1, repeat A: 301-790; exon 4 element: 9651–9950) demonstrating that this approach is successful at finding structured regions in Xist. Having defined regions where Xist is likely structured, we decided to probe full length Xist conformation in cells and use these data to constrain models of Xist conformation.

### Design of Targeted Structure-Seq

To probe the structure of Xist in cells, we adapted *in vivo* DMS chemical probing (*i.e.,* Structure-Seq [28] and DMS-Seq [26]) with targeted reverse transcription analysis and parallel sequencing, which we call Targeted Structure-Seq (Fig 1B). To ensure that Targeted Structure-Seq provides accurate, reproducible and robust DMS-sensitivity profiles for Xist (or any other RNA of interest), we set four validation criteria: (1) the DMS-reactivity profile should accurately and specifically reflect sites of DMS modification; (2) the profile for any nucleotide should be independent of the choice of RT primer used in the probing; (3) the protocol should be robust to variations in DMS treatment; (4) the approach should accurately predict accessible bases where structural information is available. A protocol that meets these criteria would provide high quality DMS-accessibility profiles that could be combined with *in silico* modeling to reveal cellular RNA conformations (Fig 1C).

### RNA modification, library construction and sequencing

**Fig 2:**
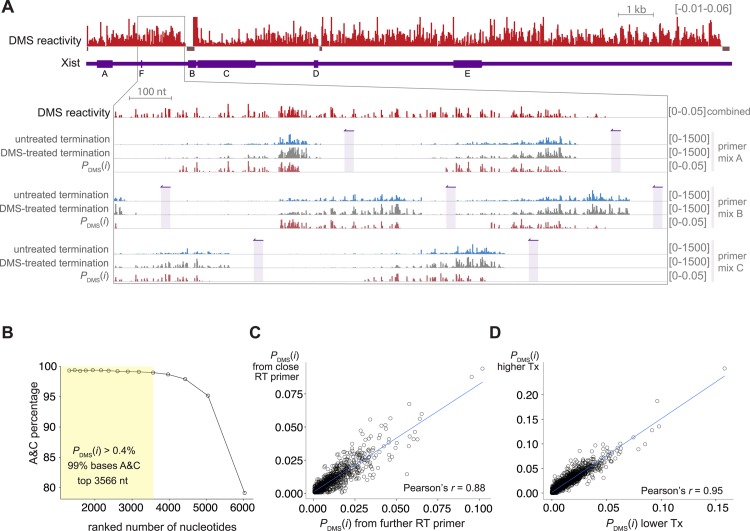
Xist DMS reactivity of A and C bases is robust and reproducible across the full length Xist RNA. **A.** The DMS reactivity profile (red) of Xist was determined by combining data from RT reactions using different primer mixtures (indicated on the right as primer mix A-C). An expanded region of Xist is shown, with primer positions indicated in purple. The probability of DMS modification, *P*_DMS_(*i*) at each A or C base (red) in each reaction is determined by comparing RT termination events in DMS-treated samples (gray) to read counts from untreated controls (blue). To better display the range of values, some positions (n = 12) have reactivity above the maximum value shown (*P*_DMS_(*i*) > 0.06). Regions with insufficient sequencing data to determine DMS reactivity are indicated with bars at *P*_DMS_(*i*) = -0.01 (n = 195, 2.3% of A and C bases in Xist). **B.** The majority of bases with high DMS reactivity are A or C. Positions were ranked by *P*_DMS_(*i*) and the percentage of bases that are A or C are plotted for the indicated positions. The yellow shaded region indicates the cutoff for bases with moderate reactivity used in this study. **C.** There is agreement between reactivity values determined using different RT primers. Each point represents an A or C base where non-zero reactivity values were determined using two different primers. For each, the reactivity derived from the farther primer (*x*-axis) is plotted against the reactivity derived from the closer primer (*y*-axis). **D.** Agreement of *P*_DMS_(*i*) values derived using two different DMS treatment conditions (slope of fit = 1.5). The lower treatment condition (*x*-axis) was 0.4% DMS, 4 min rt; the higher treatment conditions (*y*-axis) was 0.5% DMS, 8 min rt.

The conditions for DMS modification of RNA were adapted from previously developed *in vivo* DMS modification protocols [26, 28]. After isolation of cellular RNA, the sites of RNA modification on our RNA of interest (Xist) were analyzed using targeted reverse transcription. In contrast to Structure-Seq [28] and DMS-Seq [26], which probe the entire transcriptome, Targeted Structure-Seq allows focused analysis of a specific RNA. To probe the entire length of Xist lncRNA, we designed 87 Xist-specific RT primers spaced ∼200 nt apart (Fig 2A). To minimize potential interference to reverse-transcriptase elongation by RT primers hybridized to the same RNA template, we separated the RT primers into pools (with ∼600 nt between adjacent primers, Materials and Methods, S1 Data). These gene-specific primers also contained a 5’ adaptor sequence, providing compatibility with previously developed library construction protocols for sequencing [28]. Using a paired-end sequencing platform allowed us to identify both the RT start site and the RT termination site for each cDNA (Fig 1C). The RT termination site provides information about where the RT was blocked or prematurely dissociated from the RNA template. Identification of the RT start site for each read was valuable for accurate normalization of the resulting data as described below (Fig 1C).

### Determining DMS reactivity using Targeted Structure-Seq data

To probe the degree of RNA modification by DMS at each base, it is important to distinguish RT termination events caused by DMS modification (Fig 1C, red bars) from events caused by spontaneous termination (Fig 1C, blue bars). The termination events appear to have a similar distribution between the DMS-treated and untreated samples (clustering at ∼200 nt from RT start sites, Fig 2A), underscoring the need for normalization. To determine which termination events were caused by DMS, we developed a normalization pipeline inspired by similar RNA conformational analyses that use RT termination as a readout [39–41]. Our analysis pipeline allowed us to estimate the DMS reactivity of each A or C nucleotide in the RNA (*i.e.*, *P*_DMS_(*i*), the probably that the *i^th^* base will be modified by DMS) by counting termination events and read-through events in the sequencing data from the DMS-treated and untreated samples. We defined DMS reactivity as the difference between the probability of (a) termination in the DMS-treated samples and (b) spontaneous termination, determined for untreated controls (Fig 1B, Materials and Methods). We used this metric to determine reactivity scores across the full-length of Xist (Fig 2A). We assessed the quality of the resulting DMS reactivity profiles using the four criteria described above.

#### Criterion 1: DMS-modification specificity in Targeted Structure-Seq data

DMS modification inhibits RT progression only through modified A and C bases, but not G or U bases, providing a means of evaluating specificity in DMS-Seq and Structure-Seq data [26, 28]. We found that ∼99% of bases with DMS reactivity above 0.4% were A or C (3566 A and C bases, ∼40% of A and C bases in Xist, Fig 2B). By contrast, the spontaneous termination of the untreated control did not favor termination at A and C (S1A Fig). The high A + C percentage supports our conclusion that normalized data from Targeted Structure-Seq accurately identifies termination events caused by DMS modification.

#### Criterion 2: Relationship between RT primer position and observed DMS-reactivity

One concern during the design of Targeted Structure-Seq was whether the DMS-sensitivity profile would be strongly biased by the location of each base relative to the RT primers. Indeed, inspection of the aligned data prior to normalization reveals that termination events are most abundant between ∼100-400 nt from the site of the RT primer (Fig 2A and S1B–S1D Fig). The paucity of RT products shorter than 100 nt is likely caused by the loss of short fragments during library preparation (Materials and Methods), a necessary loss because attempts to capture these reads led to high levels of sequencing reads without RT products. While the raw data had an uneven distribution, we were encouraged that, after normalization, the DMS reactivity profiles were apparently even, suggesting that the profile was not substantially biased by RT position (Fig 2A).

To systematically investigate potential bias caused by RT primer location, we identified regions with reliable data from more than one RT primer (63 regions), and analyzed the relationships between data from the different RT primers (Fig 2C). DMS reactivity obtained from different RT primers was well correlated (Pearson’s *r* = 0.88, slope = 0.82), demonstrating we obtain similar reactivity data at each position when using different primers. This allowed us to evaluate DMS reactivity using the same scale across the entire 18 kb of Xist, independent of the choice of RT primers.

#### Criterion 3: Reproducibility and robustness of DMS-reactivity

We next asked whether the results of Targeted Structure-Seq were reproducible and robust. Specifically, we wanted to know whether differences in the time and concentration of DMS treatment would identify the same accessible nucleotides. Therefore we treated cells with two different DMS conditions (0.5% DMS, 8 min rt; and 0.4% DMS, 4 min rt). Performing Targeted Structure-Seq with these two treatments provided a high correlation between the DMS reactivity across A + C nucleotides (Fig 2D, Pearson’s *r* = 0.95). This analysis demonstrated that Targeted Structure-Seq provided reproducible reactivity values and was robust to differences in DMS treatment.

#### Criterion 4: Relationship between Targeted Structure-Seq reactivity and RNA structure

**Fig 3:**
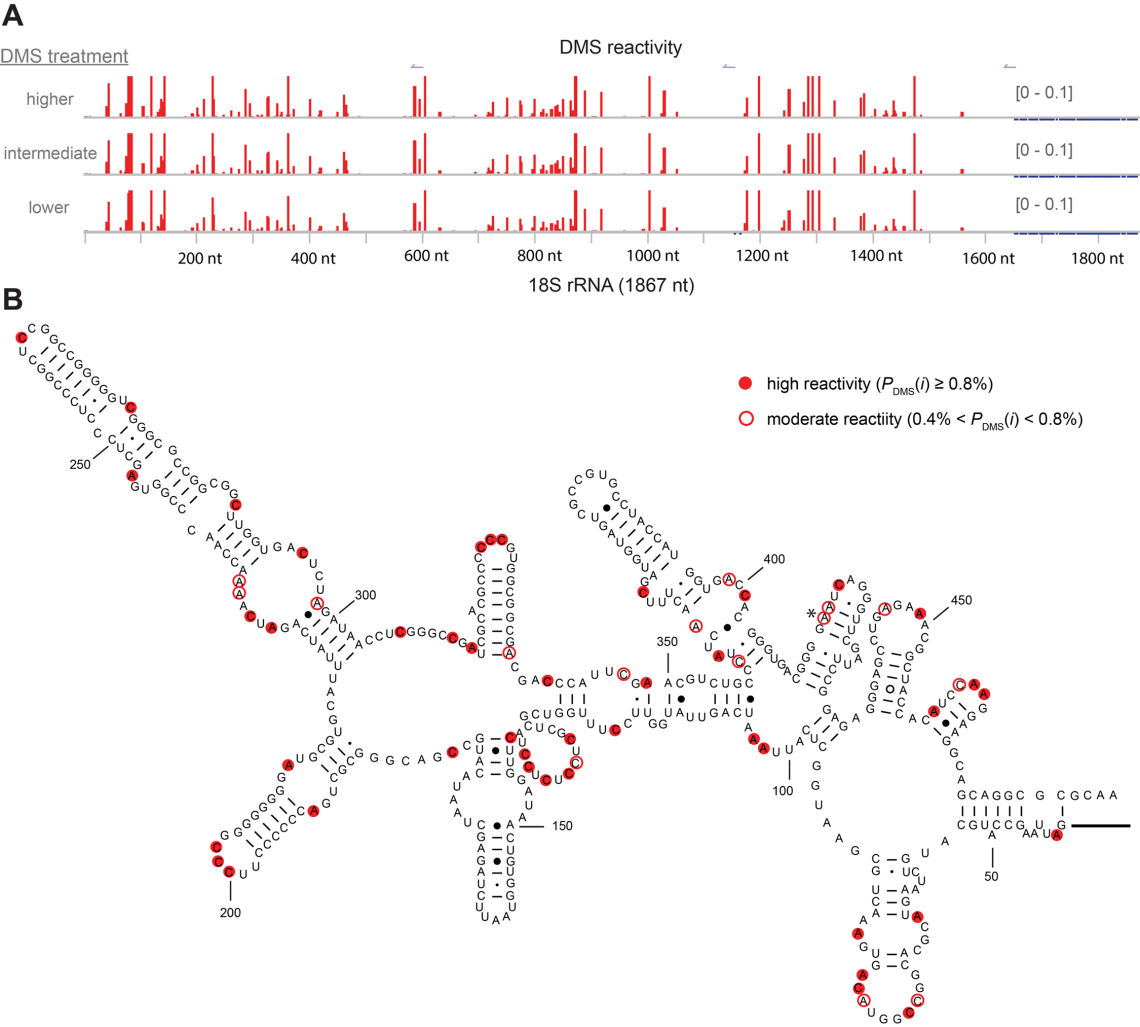
DMS reactivity accurately identifies accessible nucleotides in 18S rRNA. **A.** DMS reactivity profile (red) for A and C bases in the 18S rRNA. Data is shown for treatment with three different DMS conditions (low: 0.4% DMS v/v, 8 min; intermediate: 0.75% v/v DMS, 8 min; high: 2% DMS for 4 min. To emphasize the lower values, some positions (low, n = 17; intermediate, n = 19; high, n = 19) have reactivity above the maximum value shown (*P*_DMS_(*i*) > 0.1). **B.** The location of strongly and moderately DMS-reactive nucleotides mapped onto a model of part of the murine 18S rRNA [43]. The only conflict between the mapping data and the model in this region is indicated with an asterisk (415 nt). The data for the remainder of the structure are shown in S3 Fig.

We examined the relationship between DMS-reactivity measured by Targeted Structure-Seq and nucleotide location in an RNA structure. Previous DMS-modification and sequencing protocols have been validated by analysis of rRNA, which is sufficiently abundant to have high coverage in genome-wide sequencing analysis [26, 28, 42]. To examine the correlation between Targeted Structure-Seq reactivity and RNA structure, we determined the DMS reactivity of the murine 18S rRNA (NR_003278.3) under three different DMS treatment conditions (Fig 3) and compared these data to a modeled structure [43]. Using three 18S-specific RT primers, we were able to determine DMS reactivity across the majority of 18S rRNA (∼1500 of 1876 nt, ∼75%). Similar to what was observed for Xist, DMS-reactivity profiles across 18S rRNA were reproducible across a range of DMS conditions (S2 Fig). As expected, the DMS reactivity increased with more extensive DMS treatment (Fig 3A and S2 Fig), consistent with the similar increase that was observed in DMS reactivity for Xist (Fig 2D).

Using the intermediate DMS conditions, we identified 115 nucleotides in 18S rRNA with strong DMS reactivity (*P*_DMS_(*i*) ≥ 0.8%) and 38 nucleotides with moderate DMS reactivity (0.4% < *P*_DMS_(*i*) < 0.8%). While we focused on data from the intermediate DMS treatment, when we applied the same thresholds to data from cells subjected to higher or lower DMS treatment we identified the same nucleotides with strong DMS reactivity in all three samples, supporting our interpretation that this set of bases is most accessible to DMS alkylation in cells. We next analyzed the structural context of the modified bases.

Watson-Crick pairing generally protects A and C bases in an RNA molecule from methylation by DMS. However, the ability of A or C to react with DMS is affected by complex local environment [25]. Paired A or C bases near the end of stems, bulges, or unstable G:U pairs have some reactivity to DMS, possibly due to local instability. On the other hand, unpaired A and C bases can interact with proteins or be involved in higher-order RNA structure, rendering them inaccessible to DMS modification. Of the reactive nucleotides we identified in 18S rRNA (*P*_DMS_(*i*) > 0.4%), the vast majority (151/153, ∼99%) agreed with the structural model (Fig 3). Among the modified bases, most corresponded to unpaired bases (127/153 positions); only two of the 153 bases were flanked by Watson-Crick base pairs (Fig 3C and S3 Fig, marked by asterisks). The other modified nucleotides were: involved in non-canonical base pairing (10/153 positions), the last base of stems (9/153 positions), or adjacent to unstable G:U or G:G pairs (5/153 positions); see Fig 3C and S3 Fig).

Results from similar probing with lower or higher DMS treatment conditions led to a similar outcome (S2 Fig, 145/147 and 146/148 sites agree with predicted accessible sites in the model). The high correlation observed for the three DMS treatment conditions, together with the strong agreement between the Targeted Structure-Seq data and the conformation of the 18S rRNA, demonstrates that Targeted Structure-Seq accurately identifies bases that are accessible to modification. Having addressed our four criteria, we next used the DMS reactivity data, together with free-energy minimization, to model Xist secondary structure in cells.

### Predicting the conformation of regions in Xist

**Fig 4:**
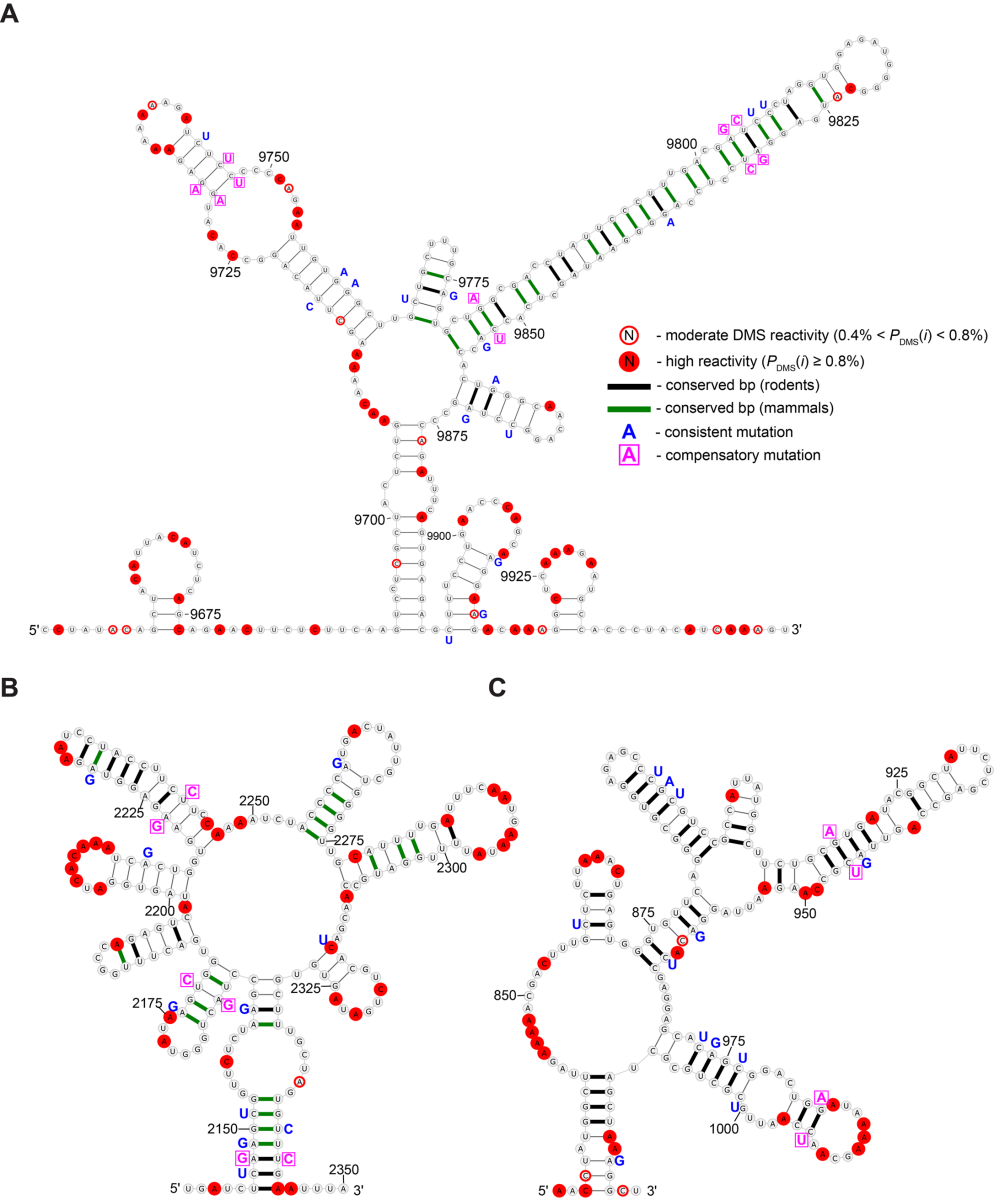
Overview and validation of three regions of Xist with structural propensity. **A.** Secondary structure model based on energy minimization constrained using DMS data from Targeted Structure-Seq results shown for the region (9651–9950 nt) that includes the previously identified conserved RNA hairpin (9779-9855) [20, 21]. The annotation key is shown to the right. **B.** The secondary structure model for 2141-2350 nt. **C.** The secondary structure model for 831-1020 nt.

**Fig 5:**
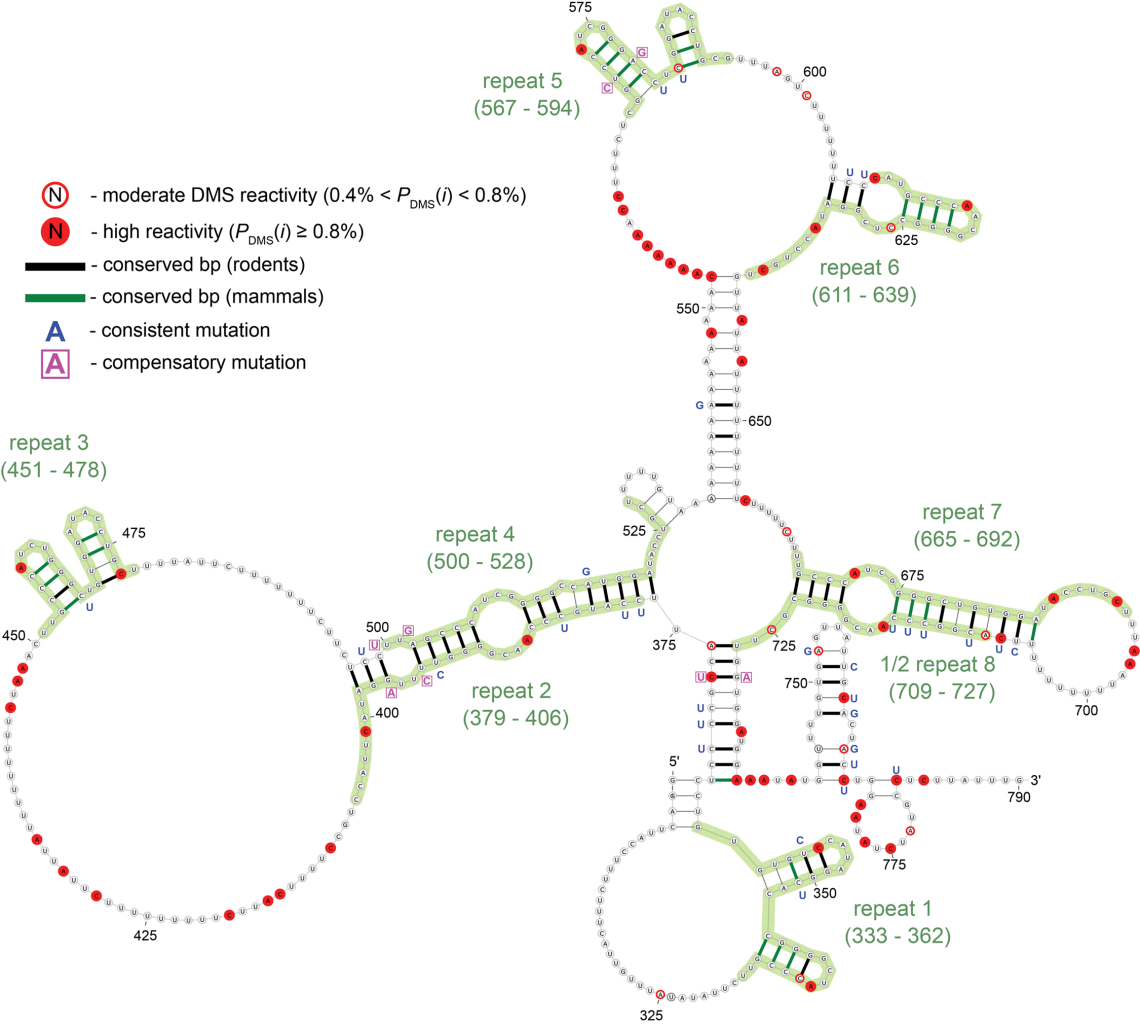
Secondary structure model for Xist Repeat A. Model based on energy minimization constrained using DMS data from Targeted Structure-Seq results. The predicted free energy ($\Delta G_{calc}^{\circ }$) is -103.5 kcal/mol for folding. The structure is annotated to show DMS reactivity, conservation in rodents and mammals, and sites of consistent and compensatory mutations—single and double point-mutations, respectively, which preserve base pairing. Annotations are defined in the key (upper left) and repeats are defined by green shading and labeled accordingly.

**Fig 6:**
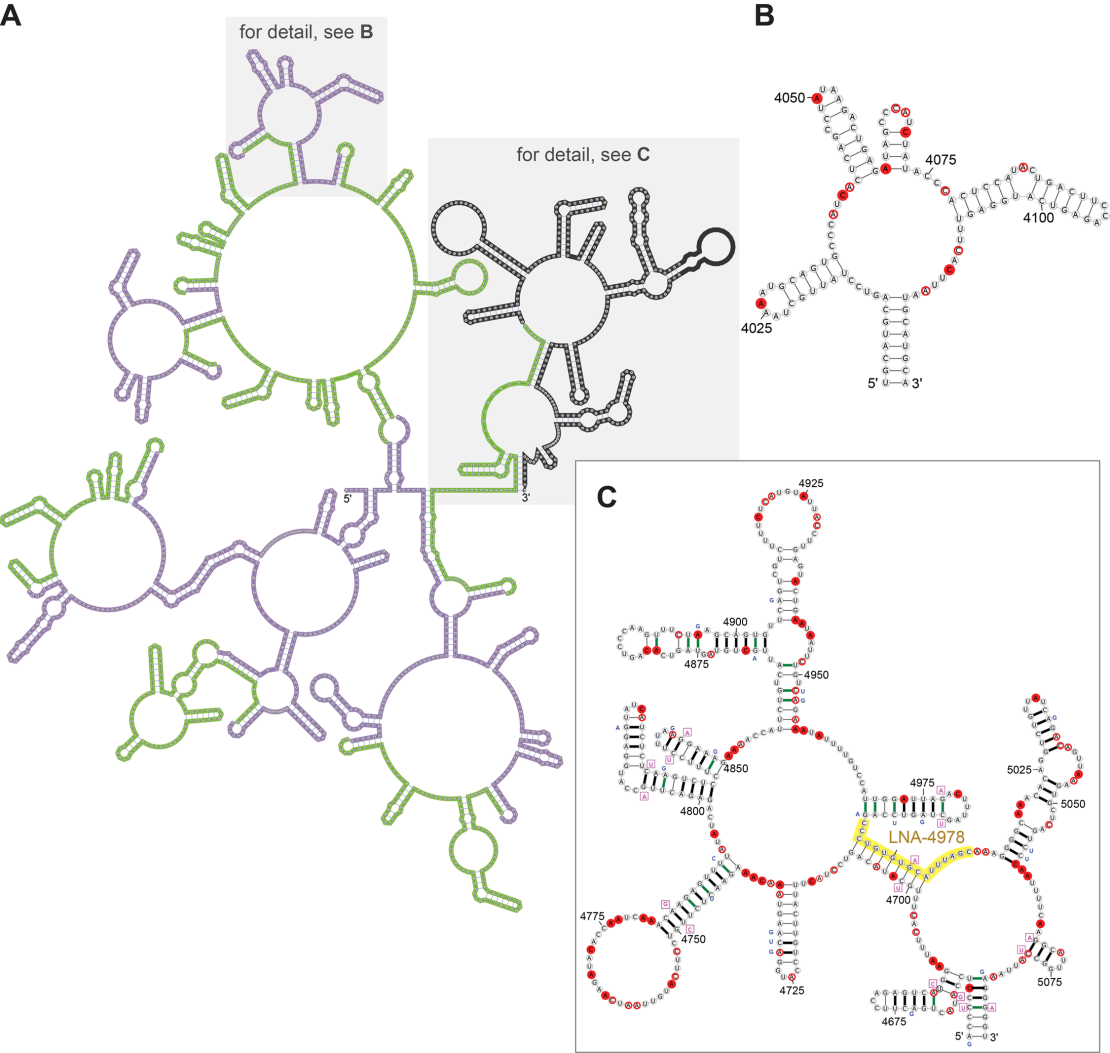
Models for RNA conformation in the region containing the Xist C repeats. **A.** A model for the region of Xist (3101–5090 nt) that overlaps the C repeats. The 14 tandem C repeats alternate in color between green and purple (to facilitate visualization). **B.** Detailed image depicting the repeated motif found in the C repeats (specifically for 4006-4125 nt) using notation as indicated in Figs 4 and 5. **C.** Similar detailed image depicting a model of the non-repetitive region 3’ to the last C repeat which shows high evolutionary conservation. The target sequence of LNA-4978, which is predicted to disrupt the structure, and knocks Xist off the chromatin, is shown in yellow. For alignment, see S36 Fig.

To build models of Xist conformation using Targeted Structure-Seq data, we performed free energy minimization using the RNAstructure software package [44]. As the RNA is modified in cells, a base that displays low reactivity is hard to interpret: it may be protected by interactions besides secondary structure (such as protein-RNA interactions). Therefore we used the most DMS-reactive nucleotides (*P*_DMS_(*i*) ≥ 0.8%) to constrain RNA secondary structure modeling, prohibiting strongly modified nucleotides from forming Watson-Crick base-pairs adjacent to Watson-Crick base-pairs. This analysis led to models for low *z*-score regions of Xist (Figs 4–6 and S4 Fig). To evaluate the quality of these models, we analyzed the locations of moderately DMS-reactive bases (0.4% < *P*_DMS_(*i*) < 0.8%), which we expected to be enriched at loops and single-stranded regions, similar to the more highly reactive nucleotides. As an independent assessment of the models, we also examined the evolutionary conservation of the predicted base pairs across rodents, and more broadly across thirteen mammals. We also evaluated instances in the models where there are consistent and compensatory mutations [45].

An example of this analysis is shown for the 9651–9950 region (Fig 4A), which includes a stem loop that overlaps with the previously identified conserved stem loop in exon 4 (9779-9855 nt) [20, 21]. The location of the highly DMS-reactive nucleotides agrees perfectly with the model (by virtue of the constraints), and the model is also strongly supported by the conservation of base pairing. In the predicted conformation of the long stem loop, most base pairs (17 of 27) are perfectly conserved in all mammals analyzed, and another seven are perfectly conserved in rodents. Mutations do occur in the hairpin; however, they maintain base-pairing, indicating evolutionary selectivity for preserving this highly stable structure ($\Delta G_{calc}^{\circ }$ = -30 kcal/mol). In addition to the long hairpin, we also found strong evidence for nearby hairpins that show high conservation in mammals (Fig 4A), demonstrating the power of this analysis to predict new RNA conformations in Xist for the other elements (Figs 4–6, S4 Fig).

### A new model for Xist repeat A conformation in cells

The Xist repeat A region is essential for gene repression [10, 11] and its structure has been previously investigated [4, 10, 18, 19]. Our analysis led to a new model for repeat A (Fig 5). As our model was constrained by probing data in cells, this represents the first model of the conformation of the Xist A repeat in cells, and differs from previously proposed models.

To test the quality of our model, we first examined the consistency between DMS reactivity and model predictions. We examined nucleotides with moderate DMS reactivity (0.4% < *P*_DMS_(*i*) < 0.8%) and found all but one (at A709; Fig 5) were consistent with the model. A bootstrapping analysis, in which free energy minimization was constrained by randomly selected subsets of DMS constraints, found essentially the same base pairs across 1000 predictions (S6 Fig, see S5–S30 Figs for similar analysis for other structured regions and S4 Data), indicating a high level of consistency between the DMS data and the model.

As an independent evaluation, we assessed conservation of base-pairing. As has been observed from examining the repeat A region of Xist in 27 mammalian species [11], it is immediately apparent that sequence conservation is localized at the repeats while the linker regions are poorly conserved (see S31 Fig). This indicates that repeats themselves, and not the sequences between each repeat monomer, may have a conserved structure and function, consistent with studies that demonstrate the repeat sequences, but not the linkers, are important for Xist function. When conservation data was mapped onto our model, we observed that base pairs within or between repeats are highly conserved (Fig 5)—most base pairs are perfectly conserved in rodents and many are well conserved across all analyzed species. Base pairs in the linker regions between A repeats are, in general, not as well conserved. For example, a predicted stem formed by a poly-A tract (536–552 nt) and a poly-U tract (640–655 nt) is comprised of sequences that are only present in murine Xist. Our observation of repeat A structural conservation is in agreement with results from previous studies demonstrating repeats of repeat A monomer are sufficient for Xist gene repression in mouse [10] and human cells [11].

In addition to the overall conservation of base-pairing, we also examined patterns of mutations (*e.g.*, when mutations occur, whether or not they preserve base-pairing). In our model, there are four compensatory mutations (double point-mutations that preserve structure) and many consistent mutations (single point mutations that preserve structure). For example, the short hairpin in repeat 5 (568–581 nt; Fig 5) is comprised of four base pairs (previously identified by Wutz *et al.*) that are perfectly conserved in all analyzed species. The base pair between U570 and A579 is identical in rodents, but in all other species (*e.g.*, humans) there is a mutation to a more stable C:G pair (Fig 5). There are two compensatory mutations in the interaction proposed to form between repeats A2 and A4 (one on each closing base pair of a 2x2 internal loop). In both base pairs, we observe a transition consistent mutation: the pair between G396 and C499 mutates to a G:U pair in primates, and shows a full compensatory mutation to an A:U pair in rabbits. There are no compensatory mutations in the hairpin formed by repeat A7 and repeat A8; however, six consistent mutations are observed on the 3’ end of the hairpin. With respect to human Xist, the majority of the murine Xist repeat A model base pairs are conserved (68 of 103, 66%). There are two fully compensatory mutations that occur at the pairs joining nucleotides 372-729 and 393-502. The latter compensatory mutation occurs in the interaction proposed for repeats 2 and 4, an interaction that has not been proposed previously, providing support for our model.

We next compared our model with those based on past predictions and *in vitro* experiments (S32–S33 Figs). Wutz *et al.* proposed the first model for repeat A in which the inverted repeats in each 26 nt monomer form two short tetraloop hairpins (12 and 10 nt, respectively; S32A Fig) [10]. NMR studies confirmed the formation of one of the tetraloops *in vitro* in the context of a single repeat and suggested the possibility of intra-repeat pairing [4, 18]. Maenner *et al.* performed extensive biochemical probing and FRET studies on an *in vitro* transcribed and annealed construct comprising the entire repeat A region of Xist using nucleases T1, T2, and V1, as well as small-molecules that included DMS [19]. Based on these data, three alternative models were proposed for different inter-repeat base-pairing patterns (hereafter referred to as Maenner models 1–3; S32–S33 Figs) [19].

Our repeat A model contains elements of both the Wutz and Maenner models. Repeats 1, 3, and 5 are essentially identical to the Wutz two hairpin intra-repeat model (Fig 5 and S32A Fig). Our model differs only in that stems in each repeat can be grown by one or two base pairs (*e.g.*, the Wutz model hairpin at nucleotides 349–358 can be extended by two additional base pairs). Our model posits a novel configuration with pairing between repeats 2 and 4, which is not found in any previously proposed structure models.

Compared with previously reported repeat A models, the conservation of our model is higher (Table 2). Each of the four repeat A models has a roughly equal number of base pairs; thus, the increased conservation of our model is due to better identification of conserved pairs rather than predicting fewer (but better conserved) base pairs. Moreover, it is notable that the predicted folding free energy of our model is more favorable than the other models (S32–S33 Figs, Table 2). For the A-repeats themselves, our new model agrees well with predictions based on free energy minimization without use of DMS constraints. Our model differs with the unconstrained model only in base pairs predicted between the 5’ and 3’ ends in the unconstrained model that include several DMS-reactive bases (see 770-783 nt, see S34 Fig) suggesting these base pairs are not present in cells and supporting our new model.

**Table 2:** Comparison of conservation and base-pairing between different models of the repeat A region of Xist.

model	$\Delta G_{calc}^{\circ }$ (kcal/mol)	bps	conservation ^*a*^	consistent^*b*^	compensatory^*c*^
Unconstrained	-129.9	110	68.3%	35	4
New (Fig 5)	-103.4	103	67.4%	33	4
Wutz refolded	-73.2	93	36.6%	10	2
Maenner model 1	-76.0	100	37.5%	16	2
Maenner model 2	-48.5	86	42.1%	26	1
Maenner model 3	-53.7	83	34.3%	10	1

See S3 Data for detailed analyses. ^*a*^ Conservation is defined here as the percentage of canonical base pairs (including GU pairs) observed in the repA sequence alignment averaged over all pairs in the model structure. ^*b*^ Number of base pairs in model with consistent mutations. ^*c*^ Number of base pairs in model with compensatory mutations.

To better understand the differences between our model and each previous model, we examined both Targeted Structure-Seq DMS data and previous probing data from *in vitro* analyses. We noticed that Maenner’s model 1, which is most similar to ours, was rejected by Maenner *et al.* because it predicted that a poly-U motif (downstream A2) was paired with a poly-A motif (downstream of A4), yet enzymatic cleavage suggested this region should be unpaired. Our model avoids this conflict because the poly-A downstream A4 is paired with poly-U downstream A6, and the poly-U motif downstream of A2 is predicted to be unpaired, consistent with the *in vitro* data reported in Maenner *et al*. [19]. In fact, when the extensive *in vitro* biochemical data were mapped onto our model, it rarely conflicted with our model (S35 Fig). There are only two bases where there are conflicts between our model and *in vitro* DMS mapping data (A348 and A516 show medium DMS reactivity *in vitro* and are located in a Watson-Crick pairs flanked by Watson-Crick pairs). Taken together, this new repeat A model is thermodynamically stable, evolutionary conserved, supported by probing of full-length Xist RNA in cells and is consistent with previous *in vitro* RNA structure probing data.

### Identification of a new conserved structural element in the repeat C region of Xist

The repeat C region of Xist is located within an extended region of predicted structure (3101–5090 nt). Notably, our model predicts that structural propensity extends beyond the last C repeat into the non-repetitive region of Xist. Compared to the average DMS reactivity throughout Xist, the reactivity was depressed across the extended repeat C region (Fig 2A). We were nonetheless able to identify some bases with high DMS-reactivity (89 out of 1613 A and C bases) and used these bases as constraints to build a model of this extended region (Fig 6).

Focusing first on the C repeats themselves, the predicted folding differs between repeats. Certain recurring structural elements are discernible, however, and there is a prototypical structural motif, comprising four recurring hairpins. These hairpins form a multibranch loop in two cases, where a basal stem forms from complementary regions on two repeats (Fig 6B). The importance of this structure across species is hard to assess: while murine Xist consists of 14 tandem repeats of repeat C, fewer copies are present in all other species that have been analyzed (including other rodents), demonstrating only modest sequence conservation [46].

Unlike the C-repeats themselves, the 441 nt subregion that includes 55 nt downstream of the last C repeat has unexpected features. This subregion produced a low *z*-score and exhibits both sequence and structural conservation in rodents (77% base pair conservation) and, to a lesser extent (60%) in other mammals (Fig 6C and S2 Data; *e.g.,* in humans, 47% of base pairs are conserved). Furthermore, in these conserved stems there are 23 consistent mutations and eight compensatory mutations. The hairpin from nucleotides 4799–4833 (containing a G:C to A:U compensatory mutation) is structurally similar to the recurring triloop predicted in repeat C (*e.g.*, nucleotides 4078–4107). Interestingly, the 150 nt window from 4731–4880 nt, which encompasses this subregion, one other triloop, as well as a large-looped hairpin, is predicted to have the second most favorable *z*-score of any Xist region (-4.05; Table 1). We have thus identified a new element in the extended repeat C region of Xist that is unusually thermodynamically stable and structurally conserved.

We reasoned that if this newly identified region 3’ of the C repeats is important for Xist function, disrupting this element would be predicted to influence Xist function in cells. Analysis of this region of Xist is complicated because previous studies in which this region is removed (as part of a much larger deletion) show little impact on Xist spreading or silencing; further, it has been proposed that there is redundancy between elements that allows Xist to spread on chromatin [10, 47]. On the other hand, as antisense oligonucleotides that target the C repeats can displace Xist from chromatin [15, 16], it is conceivable that oligonucleotides targeting this newly identified region downstream of repeat C would displace Xist from chromatin as well. Indeed, LNA-4978, which is expected to disrupt the structure of this non-repetitive region (Fig 6C, highlights, S2 Data), causes Xist to dissociate from the inactive-X chromosome [15, 16], as we have observed with high-resolution mapping experiments [7]. These experiments demonstrate the importance of this region for Xist function, and more generally illustrate how Targeted Structure-Seq can identify new structured elements that are important for lncRNA function.

## Discussion

Our knowledge of cellular RNA and its role in regulation is growing rapidly as new non-coding functions of RNA continue to be discovered [48]. Understanding the cellular conformations of these RNAs is an important area of study, and requires systematic probing of RNA structure in cells. To investigate lncRNAs such as Xist, we built upon recent progress in RNA structural probing, developing a new approach we call Targeted Structure-Seq. Using a combination of Targeted Structure-Seq and *in silico* modeling, we identified and modeled regions in Xist that show thermodynamic stability, evolutionary conservation of base-pairing, and contain many consistent and compensatory mutations. All of these features are hallmarks of conserved and functional RNA structures [45]. These models allowed us to examine the in cell conformations of novel as well as previously studied elements in Xist.

We examined the performance of Targeted Structure-Seq using four criteria. We found that Targeted Structure-Seq (1) accurately measures sites of DMS modification with high A + C to G + U specificity; (2) determines reactivity independent of the choice of RT primer used in the probing; (3) is robust and reproducible across a range of DMS conditions; and (4) accurately identifies accessible bases for the 18S rRNA. We expect Targeted Structure-Seq to have broad impact, especially as very little is known about the conformation of lncRNAs [49]. For the few full-length lncRNAs that have been systematically probed *in vitro*, such as the steroid receptor activator (SRA, 870 nt) [50], and HOTAIR [51], structural probing has provided important insight into lncRNA conformation. Targeted Structure-Seq advances these analyses by probing the RNA in cells and increasing the scalability of these experiments. Targeted Structure-Seq is well-suited to the analysis of lncRNAs such as Xist; we were able to map DMS-reactive bases across the entirety of Xist with only 164 million reads (corresponding to approximately half a lane on an Illumina 2500 HiSeq instrument). The analysis pipeline allowed us to visualize DMS-reactivity of the full length 18 kb RNA on a unified scale. For context, Xist is about twice the size of the HIV genome, which to date is the largest RNA probed *in vitro* and in virions [52]. Thus, Targeted Structure-Seq expands the scope of RNA probing approaches to include targeted analysis of mammalian lncRNAs in their cellular context.

Sequencing has another technical advantage over electrophoresis: when probing RNA by electrophoresis, it is crucial that the majority of the products from reverse transcription are correctly primed because off-target priming can interfere with analysis. With sequencing, off-target priming can be tolerated because off-target reads can be easily filtered from the aligned reads. This relaxes the stringency required for RT primers in Targeted Structure-Seq. Indeed, after alignment of the Targeted Structure-Seq reads, we found that 20-40% of paired-end reads were aligned to Xist—a huge enrichment compared to transcripitome-wide analyses (Xist represents only ∼0.07% of reads from poly-A enriched RNA-Seq reads from female MEF cells [53]). The remaining off-target reads (60-80% of reads, including those caused by mispriming of reverse transcription) did not adversely affect data analysis. Therefore, Targeted Structure-Seq dramatically enriches reads for an RNA of interest, and provides a scalable means to probe the conformation of specific RNAs, as we have demonstrated for Xist.

Using the data from Targeted Structure-Seq, we developed structural models of elements within Xist. The repeat A region of Xist is a functional element important for gene repression [10, 54, 55]. Our model of repeat A includes both intra- and inter-repeat stems (Fig 5), many of which are conserved across species. In addition, the compensatory and consistent mutations at these regions strongly indicate that these structures evolved to perform important functions. Compared to previous repeat A models, our model is significantly more thermodynamically stable and has better structure-conservation in rodents and other mammals (Fig 5, S32–S33 Figs, Table 2). Targeted Structure-Seq enabled us to develop a new model of the Xist A-repeat region that is supported by probing data, predicted stability and conservation. We were also able to examine whether the cellular environment influences the conformation of this region of Xist. Indeed, it remains unresolved how frequently RNA conformations in cells are similar to those observed *in vitro*. While differences between *in vitro* and *in vivo* conformations have been observed in certain cases (*e.g.*, see ref [26] and [28]), we found that previous data from probing repeat A *in vitro* fit our in-cell model well (S35 Fig). Thus, while it is plausible that the repeat A conformation of Xist is influenced by other protein factors *in vivo* (ATRX has been proposed to be one such factor, see [56]), the simplest interpretation of our data is that the Xist repeat A element folds to the same thermodynamically favored conformation *in vitro* and *in vivo*. This demonstrates how Targeted Structure-Seq can be used to compare differences between RNA structure *in vitro* and in cells.

Our analysis of Xist also identified several novel, evolutionarily conserved elements that are potentially functional (Fig 1A, Table 1), and we have modeled their conformations (Figs 4–6, S4 Fig). Of particular interest is the repeat C region, as it has been proposed that proteins that anchor Xist to chromatin such as YY1 [57] or hnRNP U/Saf-A [13] act at or near the C repeats. In our model, Xist repeat C is highly structured. LNAs and PNAs that displace Xist from the chromatin (LNA-C1, LNA-C2, pWS1246, pWS1248, pWS1250) [15, 16] are complementary to the multiple stem-loops predicted by our model (for hybridization sites, see S2 Data).

We also identified an evolutionarily conserved element downstream of the C repeats in Xist. Our model of this element is supported by probing data, predicted thermodynamic stability, and conservation of base pairing. Importantly, an antisense oligonucleotide targeting this novel element (LNA-4978, distinct from those that target the C-repeats) can displace Xist from chromatin [15, 16], supporting the functional importance of the elements we have identified using Targeted Structure-Seq. We expect that the new regions of Xist and their accompanying models presented here will provide the molecular framework for future studies to uncover the functions of these RNA elements.

In addition to the insight we have gained into the conformation of Xist, we have validated the utility of Targeted Structure-Seq to study lncRNA conformation in mammalian cells. We expect Targeted Structure-Seq will be particularly useful for advancing our mechanistic understanding of lncRNAs. In principle, the data here could be used to develop a conformational model of full length Xist. It remains a challenge to model the structure of an entire RNA of the size of Xist, especially due to our limited ability to predict long-range interactions. As Targeted Structure-Seq measures reverse transcription termination events, we also anticipate adapting this technology to other probing reagents (*e.g.*, SHAPE, see [58, 59]), to probe RNA structure other biological contexts, and to monitor changes across different biological conditions. These advances in RNA probing can illuminate the elements of molecular architecture from which RNA biology is built.

## Materials and Methods

### DMS treatment

Clonal female MEFs [60] were cultured in 150mm dishes with DMEM (high glucose) including 10% FBS, 2 mM glutamine, 100 U penicillin, 0.1 mg/ml streptomycin at 37°C, 5% CO_2_. Immediately before treatment, DMS was diluted to 25% in absolute ethanol, and was further diluted in PBS to desired concentration (0.4%–2% final). Hydrolysis of DMS generates acid and can depress the pH of a solution. To avoid acid-induced damage to cells, the pH of the buffer was checked after DMS treatment to ensure the reaction maintained a neutral pH. Immediately prior to treatment, MEF cells were rinsed once with PBS and were incubated in 15 ml DMS solution (or with a buffer control) at room temperature for indicated time (4-8 min). The DMS reaction was quenched by removing DMS solution and immediately rinsing the cells three times with 25 ml of 50 mM Tris pH 7.5, 100 mM NaCl, 3 mM MgCl_2_, 40 mM *β*-mercaptoethanol. Cells were collected in the same buffer using Cell Lifter (Corning) and were centrifuged at 1000g for 5 min at 4 °C. For probing of 18S rRNA, total RNA was extracted using TRIzol (Life Technologies) according to the manufacturer’s instructions. For probing of Xist, ∼5 × 10^7^ cells were lysed in 1 ml ice-cold RIPA buffer (50 mM Tris HCl, pH 8.0, 150 mM NaCl, 1% Igepal, 0.5% sodium deoxycholate, 0.1% SDS, 1 mM EDTA) supplemented with 100U SUPERasin (Invitrogen). The insoluble fraction enriched with Xist RNA was collected by centrifugation at 3000g for 1 min at 4°C, dissolved in hot TRIzol (65°C) and the RNA was purified using standard procedures.

### Gene-specific reverse transcription

TRIzol purified RNA was treated with TURBO DNase (Life Technologies) and was purified using RNeasy Mini Kit (Qiagen). For reverse transcription, a 12 *μ*l solution of 1-2 *μ*g RNA in water was annealed with pre-mixed gene-specific RT-primers (10 pmol primer total) by incubating at 70°C for 5 min, 4°C for 2 min. To this mixture, 3 *μ*l 5x first-strand buffer (5xFS buffer, 250 mM Tris-HCl, pH 8.3, 375 mM KCl, 15 mM MgCl_2_, 0.1 M DTT) was added and the solution incubated at 55°C for 10 min. While incubating at 55°C, 5 *μ*l RT pre-mix (1 *μ*l 5xFS buffer, 1 *μ*l 10 mM dNTPs, 1 *μ*l 100 mM DTT, 0.5 *μ*l Superscript III (Invitrogen), 0.2 *μ*l RNaseOut (Invitrogen), 1.3 *μ*l H_2_O) was added and kept at 55°C for 20 min for first strand cDNA synthesis. Reverse transcription was stopped by heating to 85°C for 5 min. For 18S rRNA probing, a mixture of three 18S-specific RT primers was used for reverse transcription. For Xist studies, 87 Xist-specific primers were divided and used in nine RT reactions, which were pooled later to make 3 sequencing libraries. Each RT primer includes a 5’ adaptor sequence (5’- CAGACGTGTGCTCT-3’) to facilitate library construction as previously described [28]. Primer sequences are described in S1 Data.

### Sequencing library construction

Sequencing libraries were constructed using Illumina TruSeq adaptor sequences as previous described [28] with the following modifications. Prior to linker ligation, 1 *μ*l RNase H (New England Biolabs) and 0.5 *μ*l RNase A was added to the RT reaction and incubated at 37°C for 1h to degrade RNA and purified using Ampure Xp beads (Beckman Coulter) following the supplemental protocol for miRNA enrichment by manufacturer with modifications. Briefly, RT product was mixed with 1 volume Ampure Xp beads, 3 volumes 2.5 M NaCl, 20% PEG8000, and 1 volume isopropanol and incubated at room temperature for 15 min. Ampure Xp beads were captured by magnets for 15 min, rinsed 3 times with freshly made 80% ethanol, air-dried for 5 min, and eluted in 15 *μ*l H_2_O. Purified cDNA was ligated to a 3’-adaptor (/5Phos/NNNAGATCGGAAGAGCGTCGTGTAG/3Bio/) using CircLigase (Epicentre) by mixing 6.8 *μ*l cDNA with 0.2 *μ*l 100 *μ*M 3’-adaptor, 10× buffer (0.5 M MOPS pH 7.5, 0.1 M KCl, 50 mM MgCl_2_, and 10 mM DTT), 1 *μ*l 50 mM MnCl_2_, 1 *μ*l 1 mM ATP and 8 *μ*l 50% PEG8000. The mixture was incubated at 65 °C for 2h, and then at 85 °C for 15 min to inactivate CircLigase. Ligation products were recovered by Ampure XP beads and subjected to sequential PCR as follows. cDNA was first amplified using forward and reverse primers matching 5’- and 3’- adaptor sequences (5’-CAGACGTGTGCTCTTCCGATC-3’; 5’-CTACACGACGCTCTTCCGATCT-3’) and Phusion polymerase (98°C, 20 sec; 64°C, 20 sec; 72°C, 90 sec, for 4-8 cycles; New England Biolabs). PCR products were mixed with 1.8x volume Ampure XP beads to enrich PCR products > 100 bp. Illumina TruSeq forward primer and indexed reverse primers were used for 4-8 rounds of PCR (98°C, 20 sec; 64°C, 20 sec; 72°C, 90 sec) and the final PCR product was recovered by Ampure XP beads using 1:1.8 sample:beads ratio. Multiplexed sequencing libraries were mixed and subjected to 2x75 paired end sequencing on Illumina HiSeq 2500 instruments at that Yale Center for Genome Analysis.

### Determination of DMS reactivity

Computation was performed using the Yale High Performance Computing bulldogn cluster. Sequencing reads were clipped 3 nt from 5’ end to remove NNN in the 3’-adaptor and were aligned to the cDNA sequence of the RNA of interest using bowtie2 local alignment [61]. Aligned reads with fewer than 60 nt or with more than two mismatches were discarded.

The number of RT termination events immediately 3’ of each position (relative to the cDNA, where the RT would have added the next base) were counted for both the DMS-treated samples ($n_{i}^{T}$) and the untreated samples ($n_{i}^{U}$). To assist in these calculations, a python script and example data is included (S5 Data). For each termination event, read through events for nucleotides greater than 25 nt from an RT primer (to avoid analysis of the annealing site) were counted for each base in the treated, $r_{i}^{T}$, and untreated, $r_{i}^{U}$, samples. In cases where reads aligned to multiple sites (mostly at the C repeats in Xist), the counts were divided equally among aligned sites.

We define the probably of DMS modification at the *i* ^*th*^ base as:

$$\begin{array}{c} P_{\text{DMS}}\left(i\right)\;=\;P_{\text{term}}\left(i\right)-P_{\text{spont}}\left(i\right) \end{array}$$

where

$$\begin{array}{c} P_{\text{term}}\left(i\right)=\frac{n_{i}^{T}}{r_{i}^{T}} \end{array}$$

$$\begin{array}{c} P_{\text{spont}}\left(i\right)=\frac{n_{i}^{U}}{r_{i}^{U}} \end{array}$$

The combined DMS reactivity for Xist was calculated as the average of DMS reactivity for 100-380 nucleotides from each reverse transcription primer that has >1000 read-through events. Examining the DMS reactivity for a limited range from each primer (100-380 nt) showed moderate improvement of correlation efficiency of overlapping regions compared to results without this limitation.

### *In silico* analysis of conformation regions in murine Xist

The 17918 nt murine Xist RNA sequence (GenBank Accession NR_001463) was analyzed using a 150 nt sliding window of with steps of 10 nt. Using a Perl script, each window fragment was shuffled 25 times; then, native and random sequences were folded *in silico* using the program RNAfold [34, 35]. To predict regions likely to form RNA structure, we adapted the method of Clote *et al.* using a thermodynamic *z*-score [33]. The *z*-score was calculated using the following equation:

$$\begin{array}{c} z-\text{score}\;=\;\frac{\Delta G_{\text{native}}^{\circ }-\bar{\Delta G_{\text{random}}^{\circ }}}{\sigma } \end{array}$$

where $\Delta G_{\text{native}}^{\circ }$ is the predicted native Xist Gibbs free energy of folding; $\bar{\Delta G_{\text{random}}^{\circ }}$ is the average of the shuffled “mutant” sequences; and *σ* is the standard deviation of the set of fragment sequences.

Domains likely to form RNA structure were defined by compiling overlapping windows with *z*-scores predicted to be less than 1*σ* (-2.19) more negative (favorable) than the average Xist *z*-score (-0.83). The calculation was run two additional times, yielding similar results. Sequences for these domains were extracted and used for structure modeling. In one case, in the repeat C region, two adjacent (but not overlapping) domains were joined together to form the domain for modeling. This was done because of the known repeat C annotation and because these two regions are overlapped by a window of low *z*-score (-2.02) that is close to the domain threshold.

### Secondary structure modeling

Secondary structure models were built using Gibbs free energy minimization in the software package RNAstrucure v.5.7 [44]. *In silico* folding was done at the default temperature (37°C) and using the Turner energy model [44, 62]. Calculations were guided using data obtained from Targeted Structure-Seq by constraining nt with *P*_DMS_(*i*) ≥ 0.8% (Fig 5) to be not in Watson–Crick base pairs flanked by Watson–Crick base pairs—an approach previously shown to improve prediction accuracy [62]. 2D images of predicted structures were generated using the program VARNA [63], then manually altered with the drawing program InkScape or Adobe Illustrator.

Sequences for predicted domains were queried against the GenBank database [64] using the BLASTn algorithm [65]. Identified homologous sequences were downloaded and aligned against the murine Xist predicted domain with the MAFFT alignment program [66, 67] and implementing the E-INS-i iterative refinement method [66], which is optimized for identifying conserved blocks in divergent sequences (*e.g.*, as in repeat A). Conservation of sequence and predicted secondary structure was analyzed using a Perl script that counted occurrences of predicted base pairs in the alignment (generated results in S2 Data). Models were displayed using Jalview [68]

## Supporting Information

S1 Data Excel spreadsheet listing primers and oligonucleotides used in this study. (XLSX)

S2 Data Excel spreadsheet containing a sequence alignments and structural conservation for regions of Xist referenced in the text. (XLSX)

S3 Data Excel spreadsheet containing analysis of repeat A region models that was used to generate Table 2. (XLSX)

S4 Data Excel spreadsheet containing bootstrap percentages. (XLSX)

S5 Data Python script and example data for calculating Targeted Structure-Seq stop and read though events. (ZIP)

S1 Fig Characterization of sequencing reads from Targeted Structure-Seq. **A.** The fraction of reads terminating at an A, C, G, or U base from Xist and 18S rRNA compared to the base composition of Xist and 18s rRNA. For both Xist and 18S rRNA, DMS treatment leads to an increase in termination events at A and C bases relative to composition and the termination event observed in a control that was not treated with DMS. For Xist, lower DMS: 0.4% DMS, 4 min rt; higher DMS: 0.5% DMS, 8 min rt. For 18S rRNA: lower DMS: 0.4% DMS, 4 min rt; intermediate DMS: 0.75% DMS, 8 min rt; higher DMS: 2% DMS, 4 min rt. **B-D.** Histograms representations of the distribution of sequencing read lengths for an untreated sample; **C** a sample treated with low concentrations of DMS; **D,** a sample treated with high concentrations of DMS, as described in A. Only uniquely mapped, paired-end reads with unambiguously defined insert sizes were included in this analysis.
(EPS)

S2 Fig 18S rRNA DMS reactivity profiles are reproducible across a range of DMS conditions. Pearson correlation coefficients were calculated for DMS reactivity (*P*_DMS_(*i*)) at bases A and C comparing three DMS treatment conditions (lower: 0.4% v/v DMS, 8 min; intermediate: 0.75% v/v DMS, 8 min; higher: 2% DMS for 4 min).
(EPS)

S3 Fig Correlation of DMS reactivity and the structure of 18S rRNA. DMS-reactive bases identified by Targeted Structure-seq are mapped to the structural model of 18S rRNA [43] as in Fig 3B. Highly reactive bases (*P*_DMS_(*i*) > 0.8%) are indicated with a filled red circle, moderately reactive bases (0.8% ≥ *P*_DMS_(*i*) > 0.4%) are indicated with an open circle. The DMS-reactive base flanked by Watson-Crick pairing in the model (see text) is indicated by an asterisk (415 and 1419 nt). The DMS reactivity of nucleotides after 1560 were not determined.
(EPS)

S4 Fig New elements predicted to be structured in Xist. Modeled conformations of regions of Xist are shown for **A.** 6631-7300 nt; **B.** 8201-8500 nt; **C.** 13321-13490 nt and **D.** 17461-17640 nt. Regions are annotated with reactivity, conserved base-pairings, consistent and compensatory mutations as in Fig 4.
(EPS)

S5 Fig Bootstrap analysis of the proposed model for 1–240 nt of Xist. This region was refolded 1000 times using random subsets of the strong DMS constraints identified by Targeted Structure-Seq. All base pairs shown are present in > 50% of predicted structures. Base pairs with 50–100% preserved are indicated with increasingly thick lines, and colors as indicated by the legend.
(EPS)

S6 Fig Bootstrap analysis of the proposed model for 301–790 nt of Xist. This region was analyzed as described in S5 Fig.
(EPS)

S7 Fig Bootstrap analysis of the proposed model for 831–1020 nt of Xist. This region was analyzed as described in S5 Fig.
(EPS)

S8 Fig Bootstrap analysis of the proposed model for 2141–2350 nt of Xist. This region was analyzed as described in S5 Fig.
(EPS)

S9 Fig Bootstrap analysis of the proposed model for 2551–2770 nt of Xist. This region was analyzed as described in S5 Fig.
(EPS)

S10 Fig Bootstrap analysis of the proposed model for 3101–5090 nt of Xist. This region was analyzed as described in S5 Fig.
(EPS)

S11 Fig Bootstrap analysis of the proposed model for 5301–5450 nt of Xist. This region was analyzed as described in S5 Fig.
(EPS)

S12 Fig Bootstrap analysis of the proposed model for 5661–6020 nt of Xist. This region was analyzed as described in S5 Fig.
(EPS)

S13 Fig Bootstrap analysis of the proposed model for 6031–6180 nt of Xist. This region was analyzed as described in S5 Fig.
(EPS)

S14 Fig Bootstrap analysis of the proposed model for 6231–6430 nt of Xist. This region was analyzed as described in S5 Fig.
(EPS)

S15 Fig Bootstrap analysis of the proposed model for 6441–6590 nt of Xist. This region was analyzed as described in S5 Fig.
(EPS)

S16 Fig Bootstrap analysis of the proposed model for 6631–7300 nt of Xist. This region was analyzed as described in S5 Fig.
(EPS)

S17 Fig Bootstrap analysis of the proposed model for 7981–8180 nt of Xist. This region was analyzed as described in S5 Fig.
(EPS)

S18 Fig Bootstrap analysis of the proposed model for 8201–8500 nt of Xist. This region was analyzed as described in S5 Fig.
(EPS)

S19 Fig Bootstrap analysis of the proposed model for 9351–9580 nt of Xist. This region was analyzed as described in S5 Fig.
(EPS)

S20 Fig Bootstrap analysis of the proposed model for 9651–9950 nt of Xist. This region was analyzed as described in S5 Fig.
(EPS)

S21 Fig Bootstrap analysis of the proposed model for 11071–11360 nt of Xist. This region was analyzed as described in S5 Fig.
(EPS)

S22 Fig Bootstrap analysis of the proposed model for 11721–11880 nt of Xist. This region was analyzed as described in S5 Fig.
(EPS)

S23 Fig Bootstrap analysis of the proposed model for 11921–12150 nt of Xist. This region was analyzed as described in S5 Fig.
(EPS)

S24 Fig Bootstrap analysis of the proposed model for 12331–12490 nt of Xist. This region was analyzed as described in S5 Fig.
(EPS)

S25 Fig Bootstrap analysis of the proposed model for 13321–13490 nt of Xist. This region was analyzed as described in S5 Fig.
(EPS)

S26 Fig Bootstrap analysis of the proposed model for 13651–14270 nt of Xist. This region was analyzed as described in S5 Fig.
(EPS)

S27 Fig Bootstrap analysis of the proposed model for 14321–14970 nt of Xist. This region was analyzed as described in S5 Fig.
(EPS)

S28 Fig Bootstrap analysis of the proposed model for 15131–15280 nt of Xist. This region was analyzed as described in S5 Fig.
(EPS)

S29 Fig Bootstrap analysis of the proposed model for 15431–16130 nt of Xist. This region was analyzed as described in S5 Fig.
(EPS)

S30 Fig Bootstrap analysis of the proposed model for 17461–17640 nt of Xist. This region was analyzed as described in S5 Fig.
(EPS)

S31 Fig Sequence conservation of the repeat A region of Xist. Alignment of the Xist repeat A region from 27 mammalian species adapted from Minks, *et al.* [11]. Each monomer of the A repeats marked by red boxes above. Percent identity is presented as a bar plot below the alignment. Coloring of the alignment is from white (no consensus) to dark purple (complete consensus). While the repeats themselves are well conserved, large insertions and deletions are frequently observed in the linker sequences between monomers.
(EPS)

S32 FigPreviously proposed conformations for the Xist repeat A region, together with S33 Fig.Wutz *et al.*, proposed that each monomer of repeat A forms two short hairpins based on minimal free energy prediction [10]. **S32A.** In order to compare the predicted folding energy and conservation of this model to other models, intra-repeat hairpins were fixed and remaining sequence was folded constraining the calculation with Targeted Structure-Seq data in cells. Constrained by *in vitro* RNA structure probing data, Maenner *et al.,* proposed three alternative models (S32B —S33B Figs), with model 3 (S33B Fig) as the most favored model [19]. Predicted energies ($\Delta G_{calc}^{\circ }$) were calculated using the efn2 functionality packaged with the RNAstructure program [44].(EPS)

S33 Fig Previously proposed conformations for the Xist repeat A region, together with S32 Fig. See S32 Fig.
(EPS)

S34 Fig Differences between the unconstrained and constrained models of the repeat A region. A detailed representation of the 5’ and 3’ ends of Xist is shown. The remainder of the models (blue arrows) is identical between the models and not shown. The constrained model is shown on the left (detail of same model in Fig 5). The naive, unconstrained model is shown on the right. The bases with DMS-reactivity that conflict with the naive model are highlighted with yellow. For a comparison of energies, see Table 2.
(EPS)

S35 Fig Comparison of *in vitro* probing data with a new model of the repeat A region of Xist. *In vitro* probing data by Maenner, *et al.,* [19] are mapped onto the new structure model for the Xist Repeat A region. Single-stranded (ss)RNA or weakly structured RNA, identified by DMS/CMCT chemical probing or RNase T1/T2 footprinting, are colored yellow when they overlap with Targeted Structure-Seq data. ssRNA shown in green were identified only by Maenner *et al*. Double-stranded (ds) RNA or stacked RNAs, identified by RNase V1 footprinting, are indicated by black squares.
(EPS)

S36 Fig Sequence alignment of Xist repeat C-extended region. Murine Xist 4658-5090 nt (structure model shown in Fig 6C) was aligned to corresponding sequences in 12 other species using MAFTT [66, 67] and displayed using Jalview [68]. Bases are colored according to percentage of conservation. Brackets (), paired bases; dots (.), unpaired bases; hyphen (-), gaps in sequence alignments.
(TIF)
