# Supplementary figures and images for: Probing Xist RNA Structure in Cells Using Targeted Structure-Seq
Source: PLoS Genet. 2015 Dec 8;11(12):e1005668. doi: 10.1371/journal.pgen.1005668 (PMC4672913; doi:10.1371/journal.pgen.1005668)

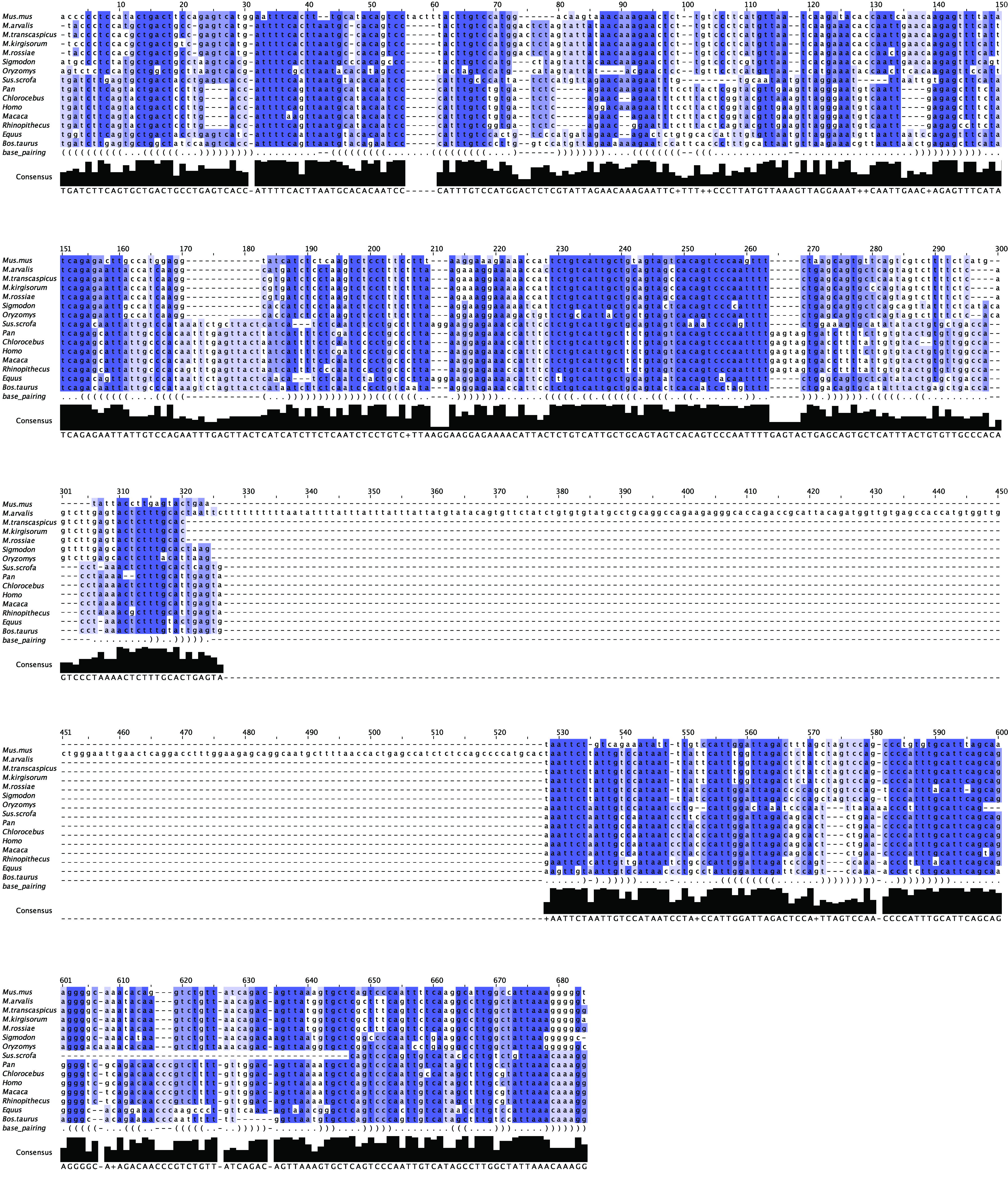

Supplement: S36 Fig — Murine Xist 4658-5090 nt (structure model shown in Fig 6C) was aligned to corresponding sequences in 12 other species using MAFTT [66, 67] and displayed using Jalview [68]. Bases are colored according to percentage of conservation. Brackets (), paired bases; dots (.), unpaired bases; hyphen (-), gaps in sequence alignments. (TIF) [file pgen.1005668.s041.tif]
